# Investigation of organophosphorus (OPs) compounds by a needle trap device based on mesoporous organo-layered double hydroxide (organo-LDH)†

**DOI:** 10.1039/d3ra01732j

**Published:** 2023-06-12

**Authors:** Razzagh Rahimpoor, Danial soleymani-ghoozhdi, Saber Alizadeh, Ali Firoozichahak, Faeze Mehregan, Razieh Firoozi

**Affiliations:** a Department of Occupational Health Engineering, Research Center for Health Sciences, School of Health, Larestan University of Medical Sciences Larestan Iran; b Student Research Committee, Faculty of Public Health, Kerman University of Medical Sciences Kerman Iran; c Department of Chemistry, Bu-Ali-Sina University Hamedan Iran; d Department of Occupational Health, Faculty of Health, Social Determinants of Health Research Center, Gonabad University of Medical Science Gonabad Iran a.firoozi@edu.umsha.ac.ir; e Medical Student, School of Medicine, Shahrekord University of Medical Sciences Shahrekord Iran; f Computer Engineering, Birjand Branch, Islamic Azad University Birjand Iran

## Abstract

Organophosphorus (OPs) compounds can endanger human health and the environment by inhibiting the acetylcholinesterase enzyme. But these compounds have been widely used as pesticides due to their effectiveness against all kinds of pests. In this study, a Needle Trap Device (NTD) packed with mesoporous organo-layered double hydroxide (organo-LDH) material and coupled with gas chromatography-mass spectrometry (GC-MS) was employed for the sampling and analysis of OPs compounds (diazinon, ethion, malathion, parathion, and fenitrothion). In this way, the [magnesium–zinc–aluminum] layered double hydroxide ([Mg–Zn–Al] LDH) modified with sodium dodecyl sulfate (SDS) as a surfactant was prepared and characterized by FT-IR, XRD, BET, and FE-SEM, EDS, and elemental mapping techniques. Then, various parameters such as relative humidity, sampling temperature, desorption time, and desorption temperature were evaluated by the mesoporous organo-LDH:NTD method. The optimal values of these parameters were determined using response surface methodology (RMS) and central composite design (CCD). The optimal temperature and relative humidity values were obtained as 20 °C and 25.0%, respectively. On the other hand, the desorption temperature and time values were in the range of 245.0–254.0 °C and 5 min, respectively. The limit of detection (LOD) and limit of quantification (LOQ) were reported in the range of 0.02–0.05 mg m^−3^ and 0.09–0.18 mg m^−3^, respectively, which shows the high sensitivity of the proposed method compared to the usual methods. The repeatability and reproducibility of the proposed method (by calculating the relative standard deviation) was estimated in the range of 3.8–10.10 which indicates the acceptable precision of the organo-LDH:NTD method. Also, the desorption rate of the stored needles at 25 °C and 4 °C, was determined to be 86.0% and 96.0%, respectively after 6 days. The results of this study proved that the mesoporous organo-LDH:NTD method can be utilized as a fast, simple, environmentally friendly, and effective method for sampling and determining OPs compounds in the air.

## Abstract 2

Schematic image of sampling and analysis of organophosphorus (OPs) compounds by organo-LDH:NTD method.

## Graphical abstract

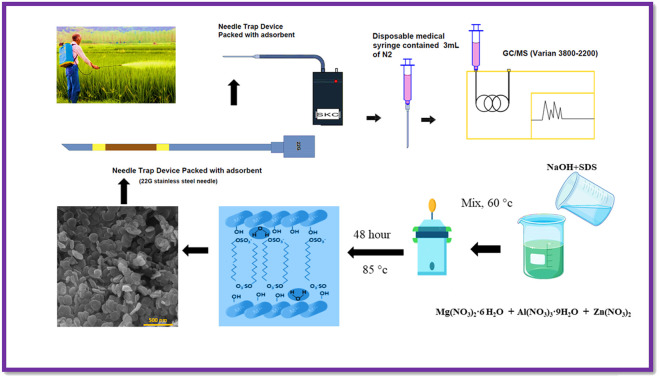

## Introduction

Organophosphorus (OPs) compounds are known as widely applied pesticides due to their favorable effect on a wide range of pests.^1^ Also, their unique characteristics such as chemical instability and high toxicity have led to the widespread use of these compounds in agriculture.^2^ These compounds can be harmful to human health and the ecosystem by inhibiting the acetylcholinesterase (AChS) enzyme.^3^ OPs compounds have significant side effects on human health, including endocrine disorders, neurotoxicity, immune system toxicity, and carcinogenesis.^4^ For example, diazinon and malathion have been classified in Group 2A and parathion in Group 2B by the International Agency for Research on Cancer (IARC).^5^

Pesticides are generally present in very low concentrations in ambient air. So, appropriate sampling and pre-concentration techniques are required to achieve high sensitivity.^6^ The National Institute for Occupational Safety and Health (NIOSH 5600) has suggested a sampling tube containing XAD-2 followed by gas chromatography for sampling and analysis of organophosphate pesticides in the air.^6^ This useful method still suffers some drawbacks such as the consumption of organic and poisonous solvents, time-consuming, and expensive apparatus.^7^

Various methods have recently been introduced to determine organophosphorus compounds. Among these methods, dispersive liquid–liquid microextraction (DLLME),^8^ solid phase extraction (SPE),^9^ magnetic solid phase extraction (mSPE),^10^ and headspace solid phase microextraction (HS-SPME)^11^ can be mentioned. One of these methods is solid phase microextraction (SPME), which has the advantages of being simple, fast, and solvent-free.^12^ However, it has disadvantages such as the limited variety of commercial fibers, short life span, and fiber fragility, as well as limited absorption capacity, which limits its use.^13^ The needle trap device (NTD) is one of the new methods of sampling and microextraction.^14–16^ This device consists of a stainless-steel needle packed with a suitable solid adsorbent, the air containing the pollutant actively passes over the adsorbent and is absorbed on its surface.^17^ This method does not have the disadvantages of the SPME method and the stages of sampling, extraction, preparation, and determination of the amount of gaseous and aqueous matrices pollutants can be done in one step, which is one of the prominent features of this method.^18^ It is worth mentioning that unlike SPME, the NTD method is not an equilibrium method and is an exhaustive method, which can be done by active sampling.

Thereby, a wide range of commercial and synthetic adsorbents have been used in NTD in past studies to determine different compounds such as MOFs, hydroxy apatite, MIPs, polymers, zeolites.^19,20^ Layered double hydroxides (LDHs) as hydrotalcite or anionic clay materials represent a new class of adsorbent materials with variable composition and chemical structure. Typically, LDHs are two-dimensional nanostructures composed of positively charged layers of metal hydroxides, charge-balancing anions, and some water molecules between the layers.^21^ These structures have the feature of anion exchange and their general formula is as follows:[M^2+^_1−*x*_M^3+^·(OH)_2_]^*X*+^·(A^*n*−^)_*x*/*n*_·*m*H_2_Owhere M^2+^ and M^3+^ are divalent and trivalent metal cations, A^*n*−^ is an interlayer guest ion with *n* valency. The values of *x* also vary between 0.22 and 0.33.^22^ LDHs are potentially good adsorbents for extraction processes due to their specific layered structure (large interlayer space), one positive charge layer on their surface, high anion exchange capacity, large surface area, and good thermal stability.^23^ LDHs have wide applications in various fields such as drug delivery,^24,25^ energy storage,^26^ absorption,^27^ electrochemical sensors,^28^ and removal of organic and inorganic pollutants from different matrices.^29^ It seems that LDHs are less effective in binding to hydrophobic organic compounds due to their strong hydrophilic surface.^30,31^ To solve this problem, surfactants are introduced as guest molecules between LDH layers to improvement hydrophobic feature of the inner surface of LDHs increasing the possibility of absorption capacity for organic pollutants.^32,33^ The anionic section of some surfactants such as dodecyl sulfate (DS^−^) anion and dodecylbenzenesulfonic acid (DBS^−^) anion have been used to modify LDHs and increase the adsorption efficiency of organic pollutants.^34^

In this study, mesoporous magnesium–zinc–aluminum-layered double hydroxide ([Mg–Zn–Al] LDH) modified with sodium dodecyl sulfate (SDS) was synthesized and characterized by FT-IR (Fourier transform infrared spectroscopy), XRD (X-ray diffraction analysis), BET (Brunauer–Emmett–Teller), Elemental mapping, and FE-SEM (Field emission scanning electron microscope) techniques. According to our information, this type of adsorbent has not been employed to determine of organophosphorus compounds (parathion, malathion, diazinon, ethion, and fenitrothion) in the air by NTD technique. Therefore, in the present study, a needle trap filled with mesoporous organo-LDH was presented to sample and analyze the organophosphorus compounds in the air.

### Instruments and pilot study

A sampling of different densities of organophosphorus compounds was done in a sampling chamber with dimensions of 15.0 × 20.0 × 30.0 cm. This glass chamber was employed for needle trap sampling, temperature monitoring sensors, and humidity control of the indoor air. To prepare the standard concentration of the studied analytes (parathion, malathion, diazinon, ethion, and fenitrothion), they were injected (all analytes together) into the chamber with a pump syringe (SP-510, JMS) at a specified injection rate. The indoor temperature of chamber was adjusted by the connected thermostat to the thermal sensor (SUN15-TI). To provide relative humidity, a hot plate and an Erlenmeyer flask containing water were used and its value was measured with a digital hygrometer (Testoterm-GmbH). The produced vapors in the chamber were diluted by a certain flow of air by an environmental sample pump (BioLite High-volume Sample Pump, SKC) and reached the desired concentration. Also, sampling of the air was done by NTD, using a personal sampling pump (222-3, SKC). For sampling by the standard method (NIOSH 5600), several solid absorbent tubes (XAD-2, 270.0 mg/140.0 mg) were purchased from SKC (USA). In this study, sampling of the analytes was done after 30 minutes of the air passage to compensate for the possible error caused by the decrease in the concentration of analytes due to settling on the surface of the chamber wall. The volume of gas has been normalized on an NTD due to the difference in ambient temperature during field testing according eqn (1).

1 *V*_1_/*V*_2_ = (*T*_1_ × *P*_2_)/(*T*_2_ × *P*_1_)

where *V*_1_ and *V*_2_ are the standard and normal gas volume (Sm^3^ and Nm,^3^ respectively), *T*_1_ and *T*_2_ are the standard and normal temperature (K), and *P*_1_ and *P*_2_ are standard and normal pressure (barA), respectively.

**Fig. 1:**
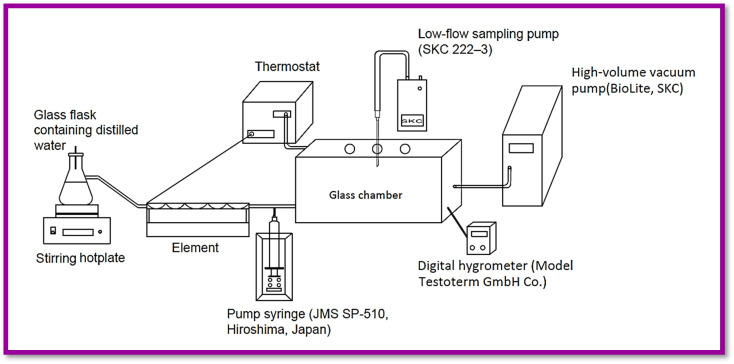
Schematic diagram of the sampling pilot.

Fig. 1 shows the schematic diagram of the designed sampling pilot.

### Chemical materials

For the synthesis of mesoporous organo-LDH nanocomposite, sodium hydroxide (NaOH-99.0%), sodium dodecyl sulfate (SDS-98.0%), magnesium nitrate hexahydrate (Mg(NO_3_)_2_·6H_2_O-99.5%), aluminum nitrate (Al(NO_3_)_3_-99.5%), and methanol (CH_3_OH-98.0%) were purchased from Sigma-Aldrich and Merck. Also, diazinon, ethion, malathion, parathion, and fenitrothion were obtained from Sigma-Aldrich. In addition, methanol (99.8%) and acetone (99.5%) were purchased from Merck to make the stock solution.

All materials were analytical grade and used without any further purification. All stock solutions were maintained at 4.0 °C. After that, functional standard solutions were prepared daily by diluting the standard solution with deionized water.

### Synthesis procedure of mesoporous organo-LDH nanocomposite

Briefly, 7.2 g of sodium hydroxide and 15.57 g of sodium dodecyl sulfate were mixed with 60.0 mL of distilled water (solution A). Also, 15.38 g of magnesium nitrate hexahydrate and 7.5 g of aluminum nitrate were mixed with 60.0 mL of distilled water (solution B). In the next step, solution B was added to the solution A drop by drop at a temperature of 60.0 °C. Then, the precipitate crystals ([Mg–Zn–Al] LDH-SDS) were washed twice with distilled water methanol. Finally, the obtained nanocomposite dried at 85.0 °C for 48 hours for evaporation of solvents. The abovementioned procedure was performed for preparation of ([Mg–Zn–Al] LDH) crystals without using of sodium dodecyl sulfate.^21^

### GC analysis procedure

The OPs compounds were analyzed with a Varian Saturn 3800 gas chromatography-mass spectrometer (GC-MS) equipped with a Varian Saturn 2200 run in EI mode detector with a Varian DB-5 column (30 m 250 μm I.D., film thickness 0.25 μm). Helium gas was used as the carrier gas with a purity of 99.999% and flow rate of 1.0 mL min^−1^. Spectroscopic detection by GC-MS included an electron impact ionization system at 70 eV, and the trap, manifold, and transfer line temperatures were set at 220, 120, and 280 degrees Celsius, respectively. The temperature programming of the column started from 80.0 °C and was held at this temperature for 2 minutes. It was then increased up to 220.0 °C at a rate of 10.0 °C min^−1^ and kept at this temperature for 2 minutes. In the next step, the column temperature was increased up to 300.0 °C and was kept at this temperature for 2 minutes. The temperature of the injection site was set in the range of 200.0–300.0 °C according to the purposes of the study. It should be mentioned that the injection of NTD into GC was done manually.

### NTD preparation method

In this study, a 22-gauge spinal needle (length 90 mm and external diameter 0.71 mm) was packed with mesoporous organo-LDH adsorbent. LDH adsorbent were mixed with glass granules to prevent clogging of the needle. Before starting of sampling, a needle was packed only with glass particles was found that the glass particles had no adsorption. Then 15 mm length of needle was packed with a mixture of adsorbent and glass granules. Also, both sides of the adsorber were filled with 3.0 mm glass wool. This act prevents the movement of the adsorbent inside the needle. A personal sampling pump was used to pass the air containing the analytes through the packaged NTD. After sampling, the NTD was connected to a medical syringe (containing three mL of pure nitrogen gas) to transfer the adsorbed analytes into the GC-MS column. The NTD was placed in the injection port of the GC-MS system for 1–7 minutes (depending on the desired desorption time). It is worth mentioning that the needles were placed in the injection site of the GC-MS machine at a temperature of 300.0 °C for 2 hours to remove interfering factors from the adsorbents inside the needles.

### Response surface methodology (RSM)

The response surface method (RSM) includes a group of mathematical and statistical techniques for modeling and analyzing problems to establish a mathematical relationship between the main parameters and the desired response. After identifying the important parameters, RMS is used to determine the optimal points. Also, this method is a good way to graphically explain the relationship between parameters and response.^35^ The response surface method based on a central composite design (CCD) was utilized to investigate the effect of factors such as temperature and time on desorption process in the GC-MS, as well as the effect of temperature and humidity factors at the sampling chamber.

### Optimization of effective parameters

Design Expert software was used to optimize affecting parameters sampling and desorption, as well as to achieve maximum efficiency. The temperature and time of desorption and the temperature and relative humidity of sampling were evaluated as affecting parameters on the performance of NTD. The concentration of analytes was set to 0.5 mg m^−3^ during the optimization process. In this study, the temperature and time of desorption were tested in the range of 200–300 °C and 1–7 min, respectively. Sampling temperature and relative humidity were also checked in the range of 20.0–40.0 °C and 25.0–70.0%, respectively.

### Method validation

Effective parameters such as limit of detection (LOD), limit of quantification (LOQ), linear dynamic range (LDR), repeatability, and reproducibility were investigated under optimal conditions for the proposed method. While there are different methods for calculating of LOD and LOQ, a signal-to-noise ratio of 3 and 10 is the most common method, respectively. LOD and LOQ were calculated by preparing low concentrations of analyzed analytes. The relative standard deviation (RSD%) was used to express the repeatability and reproducibility of mesoporous organo-LDH:NTD. Sampling was repeated five times at different concentrations using a needle to determine repeatability (in the range of 0.2 to 2.0 times the limit). Also, the reproducibility of the method was checked by sampling a fixed concentration (0.5 mg m^−3^) of the desired compounds (diazinon, parathion, malathion, ethion, and fenitrothion) using three similar needles (in terms of airflow and absorbent) and repeating five times.

### Breakthrough volume (BTV)

Determining the breakthrough volume (BTV) is essential to prevent errors during the extraction and determination of analytes. Factors such as the type of adsorbent, amount of adsorbent, concentration of analyte, and affinity of analytes can be affected on the BTV.^36^ To determine the BTV, an NTD with similar characteristics (NTD_2_) was connected to the main NTD (NTD_1_) by a PTFE connector. After sampling in optimal conditions, the desorbed analytes in NTD_2_ were used to determine BTV.

### Carryover effect

NTD desorption efficiency is one of the important issues that efforts have been made to improve it. If desorption is not complete, it results in carryover, which can be affected on the sensitivity, reproducibility, and reusability of the needle.^37^ Therefore, the desorption process should be optimized to achieve maximum efficiency. Adsorption efficiency is done by checking carryover. To determine carryover, desorption of analytes was performed twice with an interval of 2 minutes (re-injection of NTD after one sampling/desorption cycle), after sampling in optimal conditions. A comparison of the sub-peak level resulting from the second desorption with the sub-peak level resulting from the first desorption showed carryover at different times.

### Storage time

To investigation of storage feasibility of analytes in mesoporous organo-LDH:NTD, the desorption of analytes was done immediately after sampling and 1 to 6 days after sampling (storage at two temperatures of 25.0 °C and 4.0 °C). All samples were prepared under optimal conditions and at a concentration of 0.5 mg m^−3^. Both sides of the NTDs were sealed during storage at 25.0 and 4 °C.

### Measurements in a real environment

After optimization of the important parameters on the sampling and desorption of organophosphorus compounds, sampling was performed in the real environment with mesoporous organo-LDH:NTD and NIOSH 5600 methods. Finally, the measured results were compared with each other.

In this study, for the purpose of field investigation, sampling was done from the breathing zone of farmers who sprayed pistachio trees with organophosphorus pesticides.

## Results and discussion

### Characterization

In this study, the magnesium-layered double hydroxide modified with sodium dodecyl sulfate as a surfactant was prepared and characterized by FT-IR, XRD, BET, and FE-SEM techniques. FT-IR spectrometer of LDH and organo-LDH compounds are shown in Fig. S1.† The adsorption band around 3507.0 cm^−1^ originates from the OH^−^ stretching states of intralayer and interlayer water molecules. Also, appeared peak at 1638.0 cm^−1^ due to the bending state can be assigned to the presence of interlayer water molecules. The absorption band in the 1363.0–1377.0 cm^−1^ can be assigned to the *ν*3 antisymmetric state of the interlayer carbonate anions which indicates that the LDH phase contains some CO_3_^−2^ ions. Ordinary bands corresponding to SDS were observed at 2924.0, 2854.0, and 1257.0 cm^−1^ in the spectrum of the organo-LDH sample. The bands at 2924.0 and 2854.0 cm^−1^ originate from antisymmetric and symmetric C–H stretching vibrations, respectively. Furthermore, the 1257.0 cm^−1^ band is attributed to the S=O stretching vibration of the sulfate group. The appearance of these peaks confirms the presence of SDS anions within LDH layers.^38,39^

**Fig. 2:**
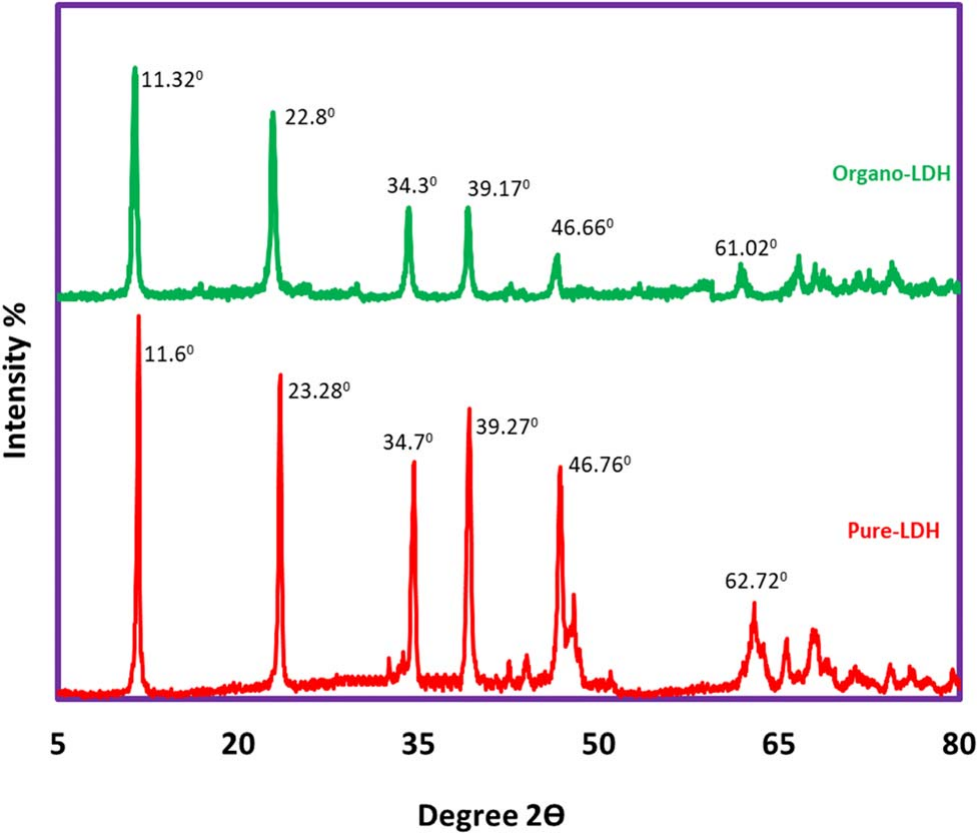
XRD pattern of pure LDH (down) and mesoporous organo-LDH (up).

Fig. 2 indicates the XRD pattern of pure LDH in the range of 2*θ*: 2.0–80.0°. The sharp and symmetrical peaks specified at 2*θ* = 11.6°, 23.28°, 34.7°, 39.27°, 46.76°, and 62.02° correspond to (003), (006), (012), (015), (018), and (113) diffraction planes, respectively that all assigned to pure LDH (down).^21^ The peaks specified at 2*θ* = 11.32°, 22. 8°, 34.3°, 39.17°, 46.66°, and 61.02° correspond to (003), (006), (012), (015), (018), and (113) diffraction planes, respectively, that all assigned to organo-LDH (up). The shifting of the characterized peaks to lower angles indicating the increase in the distance between LDH layers due to the placement of SDS anions inside the layers. These results confirm the existence of a layered structure and the accuracy of the synthesis procedure.

**Fig. 3:**
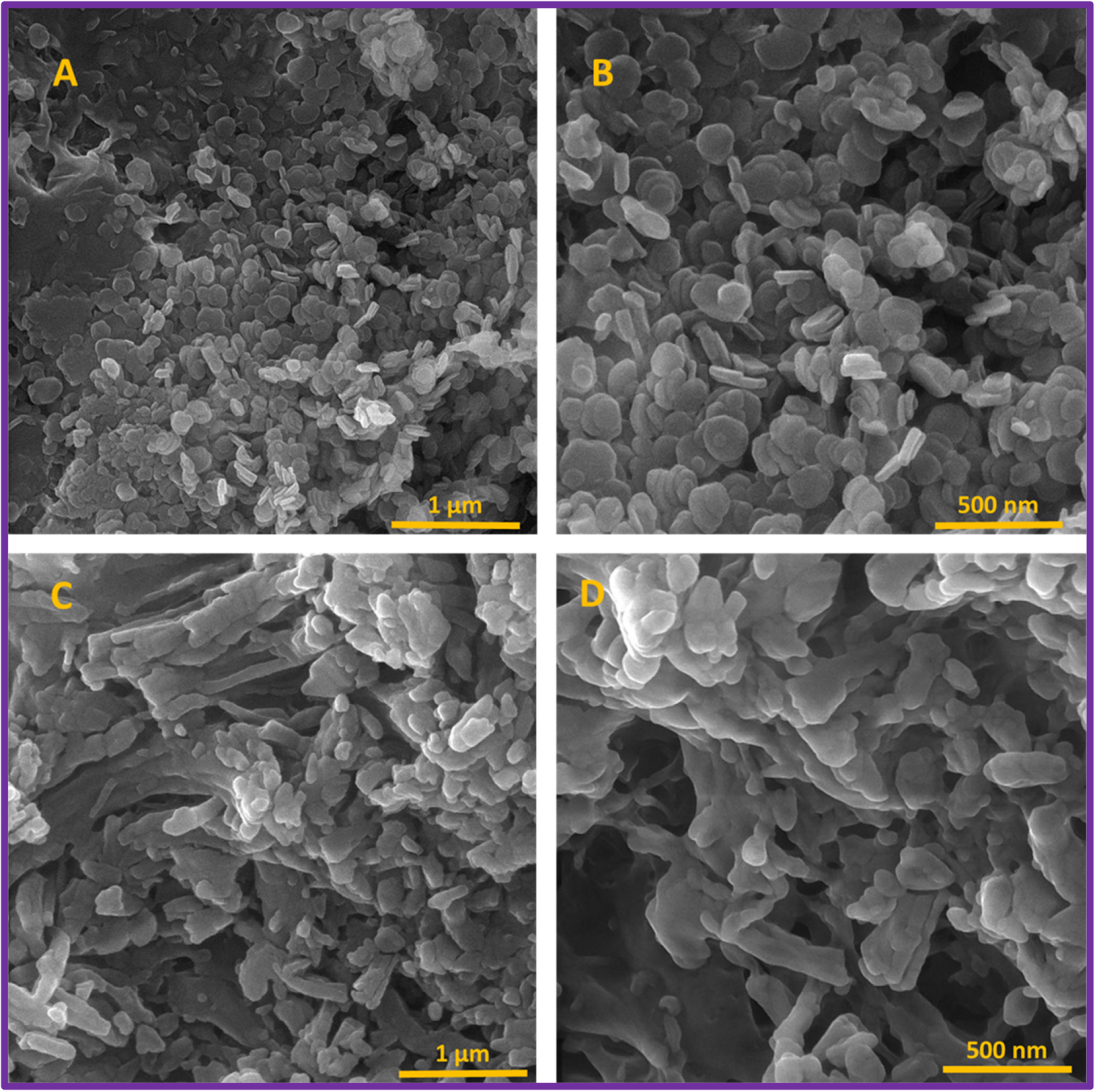
FE-SEM images of mesoporous lamellar LDH (A and B) and connected lamellar organo-LDH (C and D).

The morphology of LDH and organo-LDH was observed by FE-SEM. As can be seen in Fig. 3, the prepared LDH (up) consists of the unique, uniform, and distinguished laminarly nanosheet. Also, the organo-LDH (down) show the almost connected layered nanosheets in the presence of SDS. With SDS loading, the distinguished lamellar structure gradually disappeared and the continuously bulk phases is appeared. The reason for this change is most likely due to the placement of SDS between LDH layers. These changes are confirmed by the presented XRD results.^21^

**Fig. 4:**
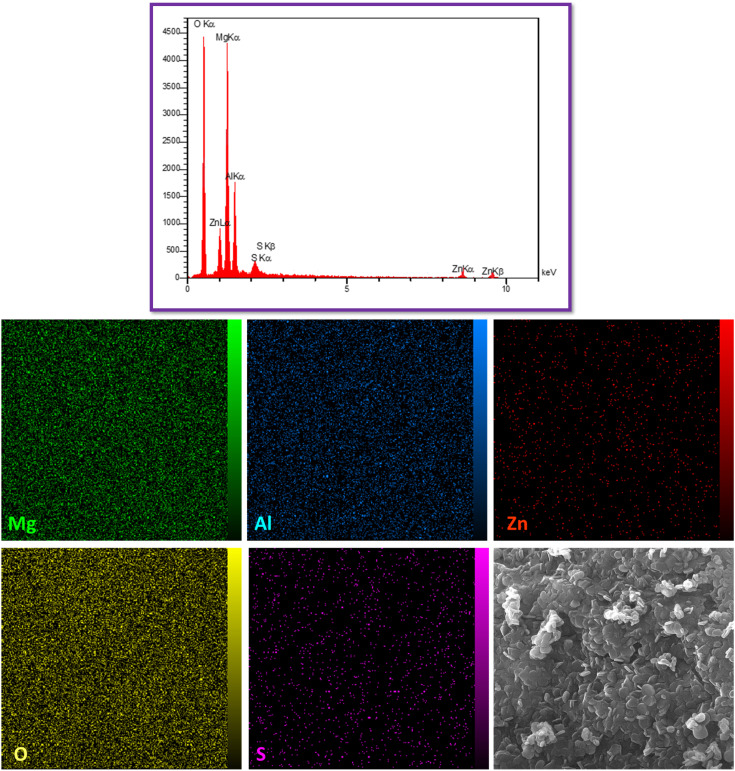
EDS pattern (up) and elemental analysis mapping (down) of mesoporous organo-LDH.

Fig. 4 presents the EDS and elemental analysis mapping of mesoporous organo-LDH nanocomposite. The elemental analysis indicates the characteristic peaks of Mg, Al, Zn in the LDH structure that approve the accuracy of LDH synthesis root. Also, the elemental analysis did not show any detectable amount of nitrogen after inclusion of dodecyl sulfate into the layer which indicating a lack of NO_3_^−^ in the interlayer. It should be noted that, the presence of S at the final structure significantly indicates replacement of the SO_4_^−2^ with NO_3_^−^ in the interlayers by dodecyl sulfate. Furthermore, the homogeneous and uniform distribution of elements proved the presence of Mg, Zn, Al, O, and S elements in the final structure.

**Fig. 5:**
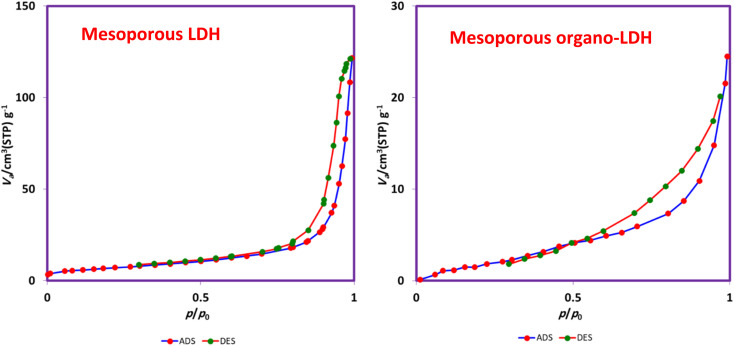
N_2_ adsorption isotherm of mesoporous LDH (left) and mesoporous organo-LDH (right).

Finally, the N_2_ adsorption isotherm of LDH and organo-LDH was recoded for evaluation of specific surface area. As can be seen in Fig. 5, the appearance of a hysteresis loop (between *p*/*p*_0_ = 0.4 and 0.8) can be assigned to a characteristic of a “type IV” isotherm, which is typical of mesoporous LDH and organo-LDH.^40^ Also, the obtained pore size distribution by the Barrett–Joyner–Halenda (BJH) technique indicates a mean pore diameter of 29.7 nm for LDH and 14.5 nm for organo-LDH. Furthermore, the calculated specific surface area (*S*_BET_) by Brunauer–Emmett–Teller (BET) method is 24.08 m^2^ g^−1^ and 10.09 m^2^ g^−1^ for LDH and organo-LDH, respectively. It is noteworthy that, the reduced surface area for LDH-SDS material illustrates the presence of SDS anions and hindrance of the exposed surface of original LDH surface by the incorporation of surfactant anion into the LDH layers for balancing of surface charge.

### Desorption parameters

**Fig. 6:**
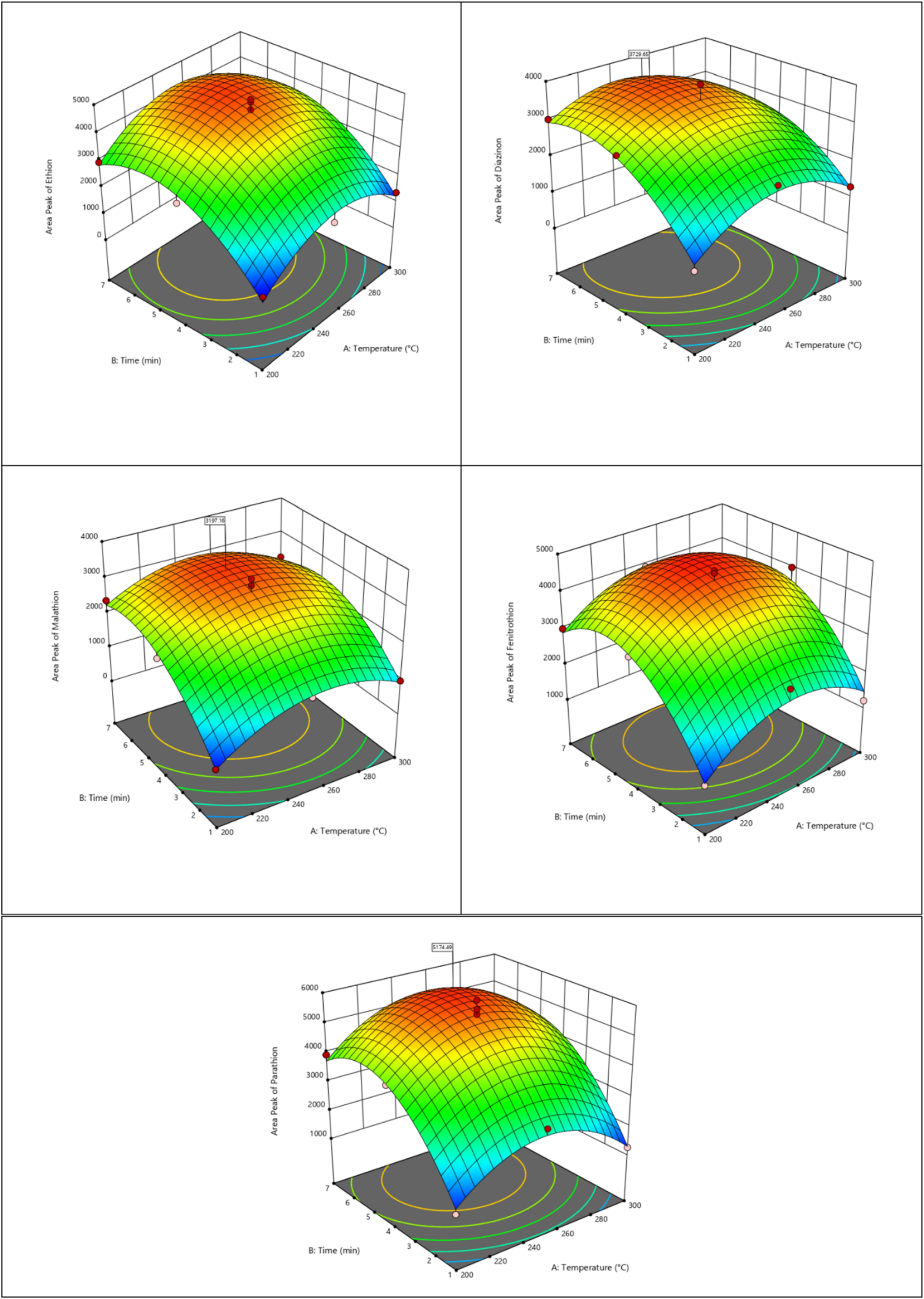
Optimization of desorption parameters of organophosphate pesticide compounds sampled with mesoporous organo-LDH:NTD.

If the desorption time and the temperature of the injection site are not well determined, the desorption process will not be performed suitably and the analytes will remain on the adsorbent and can be negatively effect on the NTD. If the desorption temperature and desorption time are high, the desorption process will be complete and fast, but it will damage the adsorbent. Therefore, finding optimal conditions is essential. For this purpose, parameters of desorption time and injection site temperature were modeled and optimized by Design-Expert software using the response surface methodology (RSM) and central composite design (CCD). In the present study, the desorption temperature was in the range of 200.0–300.0 °C and the desorption time was in the range of 1–7 minutes. Sampling was done at a concentration of 0.5 mg m^−3^. The interaction of optimal temperature and optimal time for the desorption of organophosphorus compounds (diazinon, parathion, malathion, ethion, and fenitrothion) is shown in Fig. 6. Table S1† indicates the results of optimization of desorption parameters for the targeted analytes. The optimal desorption temperature was obtained in the range of 245.0–254.0 °C. Also, the optimal desorption time was considered to be 5 minutes. Validation of the response surface model and regression coefficient was obtained by using the Design-Expert software and analysis of variance (ANOVA). The values of *R*^2^, Adj-*R*^2^, and coefficient of variation (CV) are also shown in the Table S1.† These results showed that the response were dependent on the input variables (the high value of *R*^2^) and the compatibility of the quadratic desorption model was determined by the closeness of two values of *R*^2^ and Adj-*R*^2^ to one. Also, the lack-of-fit value (*p* > 0.05) indicated that the temperature and time parameters and their mutual effects are very effective on the amount of desorption. In this model, *R*^2^ values were calculated between 0.91 and 0.96%, which indicates the appropriate response of the model.

In similar studies, the optimum conditions of desorption have been determined by SPME. For example, Saraji *et al.* used sol–gel/nanoclay composite as an adsorbent in SPME to determine of organophosphorus compounds (diazinon and parathion), and the optimum temperature and time for desorption were determined to be 270 °C and 4 min, respectively.^41^ Also, Moinfar *et al.* used MIL-53(Al)/Fe_2_O_3_ nanocomposite to extract organophosphorus compounds (diazinon, malathion, parathion) and the optimal temperature and time of desorption were obtained as 280 °C and 2 min respectively.^42^ In another study, Akbarzade *et al.* used rGOQDs@Fe adsorbent by MDSPME method to extract organophosphorus compounds (diazinon, parathion, malathion, and fenitrothion) and reported the optimum time to be 3.5 min.^43^ In another study, Saraji *et al.* used nanohybrid CNTs–SiO_2_ for the solid phase microextraction of organophosphorus compounds, and a temperature of 260.0 °C and a time of 5 min were chosen as optimal conditions for desorption.^44^ In the study of Rodrigues *et al.*, who used the HS-SPME method to determine organophosphorus compounds, the optimum temperature and time for desorption were 250.0 °C and 5 min, respectively.^45^ The comparison of desorption parameters with other studies is shown in Table S3.†

### Sampling parameters

Sampling parameters such as the temperature and relative humidity were optimized using Design Expert software and the CCD method to achieve the maximum extraction efficiency. The temperature of the sampling site was tested in the range of 20.0–40.0 °C. The results illustrated that the highest efficiency for each analyte can be obtained at a temperature of 20.0 °C. The relative humidity is another important factor that was investigated in the range of 25.0–70.0%. The results proved that the surface area for all compounds decreased with increasing humidity. This can be due to the disturbance of water vapor molecules in the adsorption of analytes on the surface of the adsorbent, which causes the active surface of the adsorbent to decrease.^19,46^ Fig. S2† shows the effect of temperature and relative humidity of sampling on the performance of mesoporous organo-LDH:NTD. The analysis of variance (ANOVA), *R*^2^ values, Adj-*R*^2^, and coefficient of variation (CV) are also shown in the Table S2† quadratic model fit was determined by the closeness of *R*^2^ and Adj-*R*^2^ values to one. *R*^2^ values calculated in this model are between 0.96–0.98%, which indicates the appropriate response of the model.

### Carryover effect

To study the carryover effect, the concentration of analytes in the sampling chamber was adjusted at 0.5 mg m^−3^. The carryover effect is affected by desorption temperature and time, which causes interference in subsequent samplings. The carryover effect as an unwanted parameter has considerable importance in reusable samplers. In this study, the results showed that the carryover effect in optimal temperature and time of desorption was less than 1.0% for all studied analytes. In a similar study, no carryover effect was observed for the determination of organophosphorus pesticides by the HS-SPME method and with ionic liquid (IL)-based silica fiber, at a temperature of 220.0 °C and a time of 10 min.^11^ Also, in Ahmadkhaniha's study, the results showed that no memory effect occurred at a temperature of 230.0 °C and a time of 4 min.^47^

### Breakthrough volume investigation

Breakthrough (BT) occurs when the adsorbed analytes in NTD_2_ is more than 10.0% of the adsorbed analytes in NTD_1_. In the present study, the analyte concentration in the sampling chamber was 0.5 mg m^−3^ to determine BTV. The results indicated that by increasing the sampling time up to 10 hours, the surface area of the analytes increased, but BT was not observed. According to the flow rate of air passing through the NTD (3.0 mL min^−1^), after 10 hours of sampling, 1800.0 mL of air has been passed through the adsorbent surface. Therefore, 0.9 μg of each analyte has been adsorbed.

### Repeatability and reproducibility

Evaluation of repeatability and reproducibility was done in the concentration range of 0.2–2.0 times the permissible limit and 0.5 mg m^−3^, respectively, under optimal conditions. The results of repeatability and reproducibility of the desired method using RSD% are summarized in Table 1. The results illustrates that the relative standard deviation for the studied compound in different concentrations was in the range of 3.8–9.3 and also for three different NTDs, it was between 6.9 and 10.1. In the study of Akbarzade *et al.*, the relative standard deviation was in the range of 3.9–8.6.^43^ Also, in the study of Saraji *et al.*, repeatability was reported in the range of 5.1–6.3 and 5.9–7.8.^44^ These results proved that the proposed sampling and analysis system in this research has acceptable accuracy.

**Table 1:** Reproducibility and repeatability of mesoporous organo-LDH:NTD for sampling and analysis of organophosphate pesticides compounds

Analyte	Repeatability		Reproducibility		LOD (mg m^−3^)	LOQ (mg m^−3^)	LDR (mg m^−3^)
	Concentrations (mg m^3^)	RSD%	NTD	RSD%			
Diazinon	0.02	8.40	NTD_1_	8.10	0.04	0.14	0.14–1500.0
	0.05	9.20					
	0.10	5.30	NTD_2_	9.30			
	0.15	6.40					
	0.20	9.10	NTD_3_	8.80			
Fenitrothion	0.02	6.10	NTD_1_	9.20	0.04	0.13	0.13–1500.0
	0.05	7.20					
	0.10	9.20	NTD_2_	7.10			
	0.15	8.60					
	0.20	3.90	NTD_3_	9.30			
Malathion	2.00	5.60	NTD_1_	9.10	0.02	0.09	0.09–1500.0
	5.00	6.60					
	10.00	3.80	NTD_2_	7.60			
	15.00	6.90					
	20.00	5.90	NTD_3_	6.90			
Parathion	0.02	8.20	NTD_1_	9.30	0.05	0.18	0.18–1500
	0.05	9.10					
	0.10	4.10	NTD_2_	10.10			
	0.15	6.70					
	0.20	9.30	NTD_3_	8.60			
Ethion	0.08	8.20	NTD_1_	10.1	0.02	0.10	0.10–15000
	0.20	6.10					
	0.40	5.20	NTD_2_	9.80			
	0.60	7.30					
	0.80	8.30	NTD_3_	8.90			

### Method validation

The performance of NTD packed with mesoporous organo-LDH adsorbent was investigated to determine the number of organophosphorus compounds in optimal conditions, according to various analytical parameters such as LOD, LOQ, LDR. Table 1 presents LOD, LOQ, and LDR values for sampling and analysis of organophosphorus compounds. In this study, the external calibration curve was drawn in the range of 0.02–1500 mg m^−3^. Also, the linearity of the calibration curve was determined according to the correlation coefficients (*R*^2^: 98–99). The value of LOD in NIOSH 5600 for sampling of diazinon, parathion, ethion, and malathion are reported as 0.04, 0.04, 0.04, and 0.1 μg in the sample, respectively.^6^ In Bagheri *et al.*'s study, the immersed SPME method was used to sampling of organophosphorus compounds, and the LOD value was reported in the range of 0.05–1.0 ng mL^−1^.^48^ Also, in the study of Rodrigues *et al.*, LOD and LOQ values were reported in the range of 2.16–10.85 and 6.5–32.9 μg L^−1^, respectively.^45^ According to these results, NTD packed with organo-LDH adsorbent showed high sensitivity to determine OPPs. Also, the ability of the proposed method is compared with several other methods in Table 2.

**Table 2:** Comparison of mesoporous organo-LDH:NTD with other techniques for determination of organophosphorus pesticides

Technique	Determination	Matrix	LOD	LOQ	RSD (%)	Ref.
NIOSH 5600						
HS-SPME	GC-MS	Apple	0.01–0.2 (μg kg^−1^)	0.05–1.0 (μg kg^−1^)	0.1–13.7	49
HS-SPME	GC-MS	Water	20.0–35.0 (ng L^−1^)	—	8.0–10.0	50
DI-SPME	GC-CD-IMS	Water	5.0–20.0 (ng L^−1^)	10.0–50.0 (ng L^−1^)	5.1–6.3	44
SPE cartridges	GC-NPD	Air	0.01–0.03 ng mL^−1^	—	4.5–8.5	51
Sorbent tube	GC-NPD	Air	0.04–0.3 μg m^−3^	0.1–1.0 μg m^−3^	4.0–11.0	52
	GC-MS	Air	0.02–0.15 μg m^−3^	0.06–0.5 μg m^−3^		
NTD	GC-MS	Air	0.02–0.05 ng mL^−1^	0.09–0.18 ng mL^−1^	3.8–9.2	Current study

### Storage time

The results of the evaluation showed that the recovery rate of samples stored at 25.0 °C reached to 86.0% after 6 days. Also, the results of the desorption of the samples stored at 4.0 °C showed that the resumption rate is about 96.0%. According to NIOSH 5600, the recommended storage time for organophosphorus is 0.0 and 25.0 °C for 10 and 29 days, respectively.

### In-field measurements

In the last stage of evaluation, the ability of the proposed method in measuring of organophosphorus compounds was examined in real conditions. For this purpose, several real samples were taken from the environment by using mesoporous-organo-LDH adsorbent (spraying by farmers). The results of measuring organophosphorus compounds with NIOSH 5600 and mesoporous organo-LDH:NTD methods are shown in the Table 3 (with 5 repetitions).

**Table 3:** The results of sampling and analysis of organophosphorus compounds in real environment with mesoporous organo-LDH:NTD and NIOSH 5600 method

Mesoporous organo-LDH:NTD			NIOSH 5600		Percentage difference
Analyte	Concentration (mg m^−3^)	RSD%	Concentration (mg m^−3^)	RSD%	
Diazinon	0.12	6.9	0.09	5.3	25.00%
Fenitrothion	0.09	7.4	0.07	8.1	22.2.0%
Malathion	9.12	6.9	7.21	5.8	21.92%
Parathion	0.08	8.2	0.06	8.9	25.00%
Ethion	0.21	7.1	0.17	7.3	19.04%

According to the obtained results, there was no significant difference (*p* value > 0.05) between these two methods (NIOSH 5600 and mesoporous organo-LDH:NTD) for field sampling of the desired analytes. These results showed that the developed NTD can be used as a new, solvent-free and rapid method for sampling and analyzing of organophosphorus compounds in work environments.

## Conclusion

In this try, mesoporous organo-LDH nanocomposite was synthesized and employed for the first time as an adsorbent for the sampling and analysis of OPs compounds using NTD-GC-MS method. The affecting parameters on the NTD were optimized using design expert software. By utilizing the proposed method in the laboratory, OPPs were sampled and quantified with good recovery and acceptable RSDs. Also, the performance of the proposed method was investigated by sampling and analyzing the desired analytes in the real environment. The results showed that mesoporous organo-LDH is a suitable candidate as an adsorbent for sampling of OPPs compounds. Among the advantages of the mesoporous organo-LDH:NTD-GC-MS method, we can point out acceptable repeatability and reproducibility, very high BTV, low carryover, low LOD and LOQ, simple processes and also no use of organic solvent.

Finally, the obtained results show that the proposed system can be used as a simple, solvent-free, efficient and versatile method for the extraction and determination of organophosphorus pesticides in public-occupational health, and environment. It should also be introduced as an alternative to the usual and old methods.

## Conflicts of interest

The authors declare no conflict of interest regarding the publication of this article.

## Supplementary Material

RA-013-D3RA01732J-s001
