# Supplementary material for: Investigation of organophosphorus (OPs) compounds by a needle trap device based on mesoporous organo-layered double hydroxide (organo-LDH)
Source: RSC Adv. 2023 Jun 12;13(26):17656–66. doi: 10.1039/d3ra01732j (PMC10258888; doi:10.1039/d3ra01732j)
Supplement: RA-013-D3RA01732J-s001 [file RA-013-D3RA01732J-s001.pdf]

Electronic Supplementary Material (ESI) for RSC Advances.
This journal is © The Royal Society of Chemistry 2023

Investigation of Organophosphorus (OPs) Compounds by Needle Trap Device Based on the Mesoporous Organo-Layered Double Hydroxide (Organo-LDH)

Razzagh Rahimpoor[1], Danial soleymani-ghoozhdi[2], Saber Alizadeh[3], Ali Firoozichahak[*4], Faeze Mehregan[5], Razieh Firoozi[6]

[1]Department of Occupational Health Engineering, Research Center for Health Sciences, School of Health, Larestan University of Medical Sciences, Larestan, Iran

[2] Student Research Committee, Faculty of Public Health, Kerman University of Medical Sciences, Kerman, Iran

[3]Department of Chemistry, Bu-Ali-Sina University, Hamedan, Iran.

[4] Department of Occupational Health, Faculty of Health, Social Determinants of Health Research Center, Gonabad University of Medical Science, Gonabad, Iran

[5] Medical student, school of medicine, Shahrekord University of Medical Sciences, Shahrekord, Iran.

[6]Computer Engineering, Islamic Azad University, Birjand branch. Birjand, Iran.

**Corresponding author:*

Ali firoozichahak, Faculty Member, Department of Occupational Health, Faculty of Health, Gonabad University of Medical Sciences, Gonabad, Iran.

Email: ***a.firoozi@edu.umsha.ac.ir***

Table S1. Response Surface Quadratic desorption model of Organophosphate pesticides by Mg-LDH: NTD

**Parameters/analytes**	**Diazinon**	**Ethion**	**Fenitrothion**	**Malathion**	**Parathion**
Optimal Temperature (◦C)	244.922	253.199	254.014	251.136	251.944
Optimal Time (min)	5.408	5.132	4.973	5.374	5.261
R-Squared	0.9188	0.9676	0.9615	0.9632	0.9657
Adj R-Squared	0.8609	0.9445	0.9340	0.9369	0.9412
SD	350.67	308.84	306.74	230.41	379.32
CV	12.58	9.08	9.14	10.15	9.13
PRESS	3.946E+06	3.008E+06	3.590E+06	1.959E+06	7.070E+06
Lack of Fit	0.5400	0.3469	0.3338	0.2291	0.1437
p-value	0.0011	< 0.0001	< 0.0001	< 0.0001	< 0.0001

Table S 2. Response Surface Quadratic sampling model of Organophosphate pesticides by Mg-LDH: NTD

**Parameters/analytes**	**Diazinon**	**Ethion**	**Fenitrothion**	**Malathion**	**Parathion**
Optimal Temperature (◦C)	20.000	20.580	20.000	20.152	20.185
Optimal Humidity (%)	25.000	25.291	25.000	25.852	25.812
R-Squared	0.9839	0.9746	0.9594	0.9617	0.9612
Adj R-Squared	0.9724	0.9565	0.9305	0.9343	0.9335
SD	220.07	274.97	283.58	241.19	242.69
CV	7.10	9.22	11.73	9.71	9.76
PRESS	1.740E+06	1.534E+06	2.499E+06	2.018E+06	2.054E+06
Lack of Fit	0.3180	0.8249	0.3307	0.3535	0.3459
p-value	< 0.0001	< 0.0001	< 0.0001	< 0.0001	< 0.0001

Table S 3: Comparison of desorption parameters organo-LDH: NTD with other techniques for determination of organophosphorus pesticides

Technique	Optimal desorption time (min)	Optimal desorption temperature (°C)	Ref
SPME- sol-gel/nanoclay	4	270	1
SPME- MIL-53(Al)/Fe2O3	2	280	2
MDSPME- rGOQDs@Fe	3.5	NR	3
SPME -CNTs-SiO2	5	260	4
HS-SPME	5	250	5
HS-SPME	5	260	6
HS-SPME	NR	NR	7
NTD	4.9-5.4	244-254	Current study

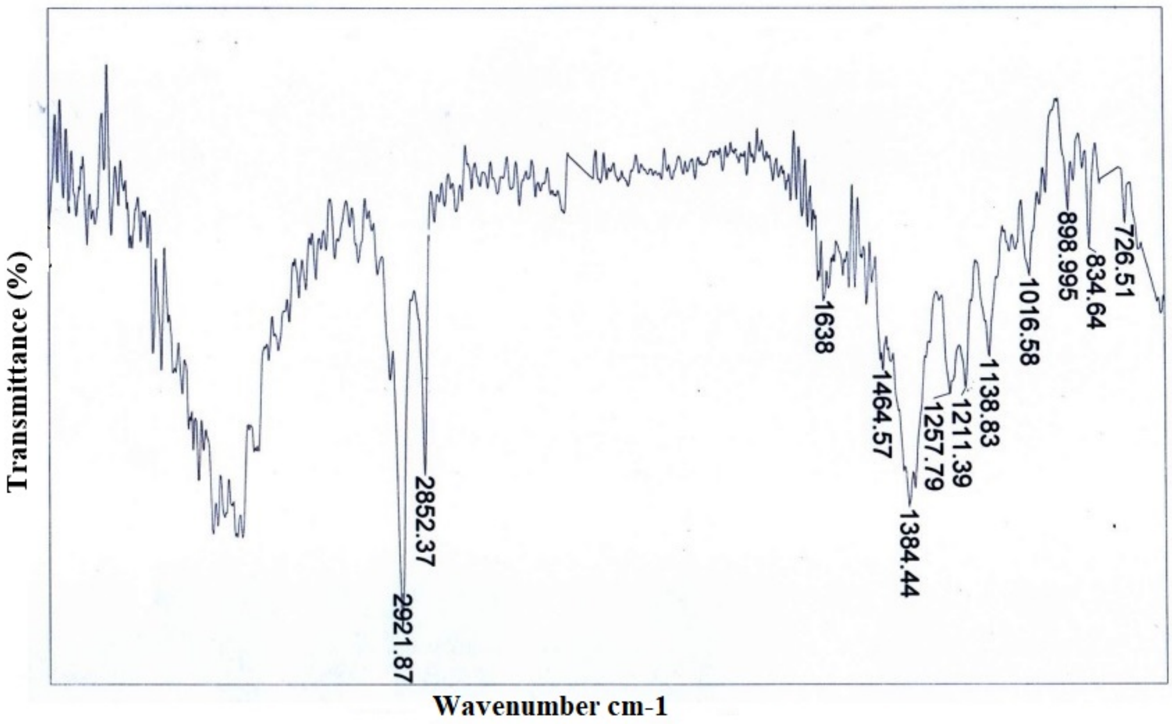

Fig. S 1: FT-IR spectroscopy of mesoporous organo-LDH

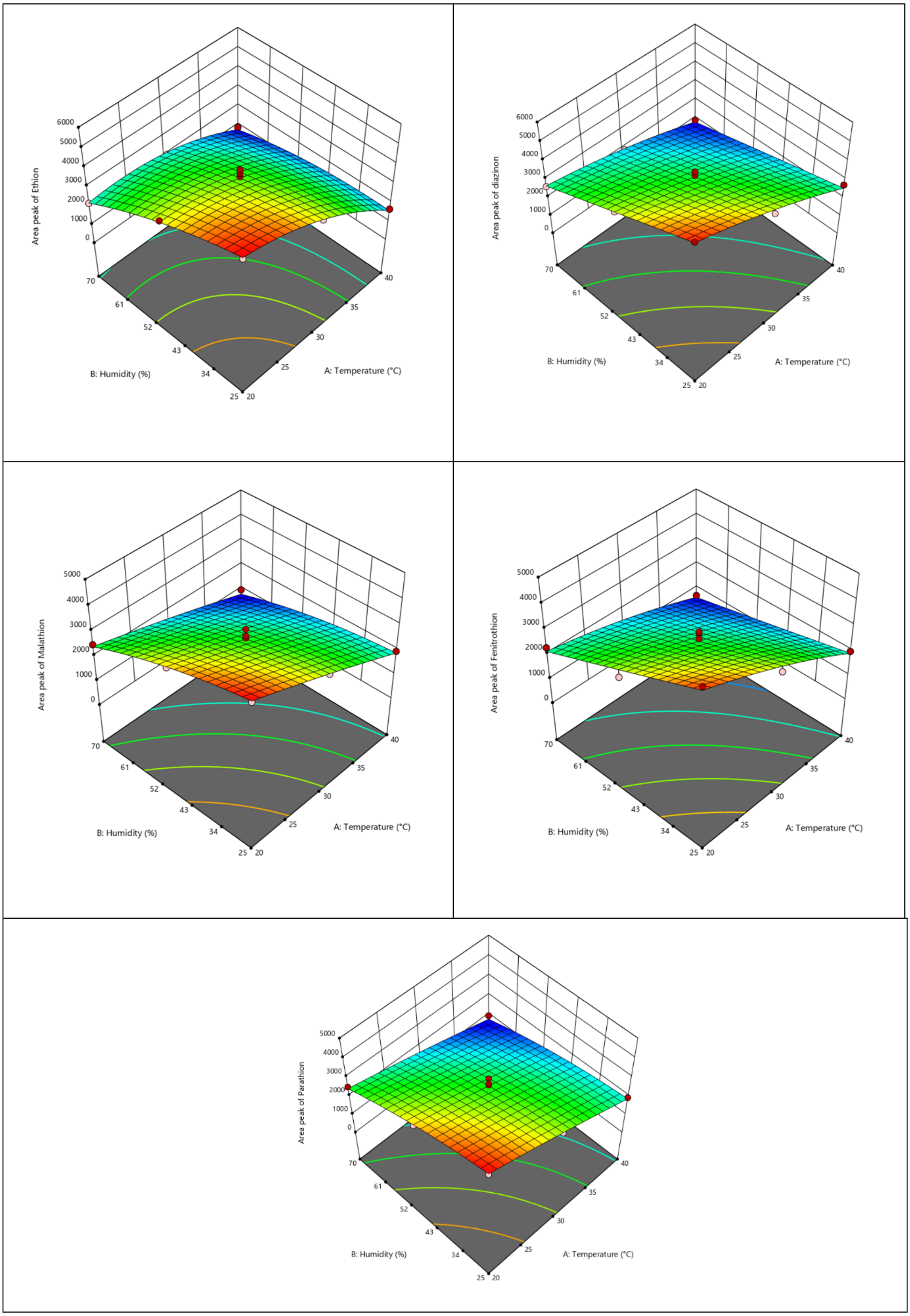

Fig. S 2: Optimization of sampling parameters of Organophosphate pesticides compounds sampled with NTD: Mg-LDH

Reference

1 M. Saraji, M. T. Jafari and H. Sherafatmand, *Anal. Bioanal. Chem.*, 2015, **407**, 1241–1252.

2 S. Moinfar, A. Khodayari, S. Sohrabnezhad, A. Aghaei and L. A. Jamil, *Microchim. Acta*, 2020, **187**, 647.

3 S. Akbarzade, M. Chamsaz, G. H. Rounaghi and M. Ghorbani, *Anal. Bioanal. Chem.*, 2018, **410**, 429–439.

4 M. Saraji, M. T. Jafari and M. Mossaddegh, *J. Chromatogr. A*, 2016, **1429**, 30–39.

5 F. de M. Rodrigues, P. R. R. Mesquita, L. S. de Oliveira, F. S. de Oliveira, A. Menezes Filho, P. A. de P. Pereira and J. B. de Andrade, *Microchem. J.*, 2011, **98**, 56–61.

6 L. B. Abdulra'uf and G. H. Tan, *Food Chem.*, 2013, **141**, 4344–4348.

7 D. A. Lambropoulou and T. A. Albanis, *J. Chromatogr. A*, 2001, **922**, 243–255.
